# Artificial intelligence to shed light on the algal dark proteomes

**DOI:** 10.1016/j.patter.2025.101421

**Published:** 2025-11-14

**Authors:** Maxence Plouviez, Eric Dubreucq

**Affiliations:** 1 Cawthron Institute, 98 Halifax Street East, Nelson, New Zealand; 2 IATE, Institut Agro – INRAE – Montpellier University, Montpellier, France

## Abstract

Nelson et al. developed LA⁴SR, a large-scale language-model framework to classify translated open reading frames (ORFeomes) across ten algal phyla. LA⁴SR achieves near-complete protein classification coverage even for algal dark proteomes, where alignment tools often return “no hit.” The framework provides a leap forward for high-throughput protein classification in algae and could be a promising tool for better classifying proteins from many organisms.

## Main text

**Known unknowns**: omics technologies have significantly accelerated scientific discovery in biological research, but most importantly, they have redefined what we know about the genome’s encoding potential. For instance, ribosome profiling led to the discovery that ribosomes are making proteins that map to “unexpected” regions of the genome initially not thought to produce proteins (i.e., noncanonical open reading frames [ORFs]).^1^^,^^2^^,^^3^ From microbes to humans, genetic information is therefore encoded with a greater density than previously suspected, and the proteome (i.e., the entire set of proteins from an organism) exhibits a level of complexity far exceeding earlier expectations.^1^^,^^3^^,^^4^ Unfortunately, many proteins remain “unknown,” or “dark,” because they lack detectable homologs in existing databases, meaning structural information or functional annotations are lacking.^4^^,^^5^ The expanse of this “dark proteome” is considerable, as metagenomics projects have revealed that 17% (∼1.7 billion) of non-redundant microbial protein sequences from across the biosphere do not harbor homologs in any of the >100,000 available reference genomes.^5^ For lesser-studied organisms like algae, the dark part could represent 65% of the whole proteome.^6^ Scientists are only beginning to understand the role of these unknown proteins in cellular function and in responses to, for example, diseases in mammalian cells.^2^^,^^3^ Consequently, what biological information is held in the dark proteomes?

**The challenge**: classical alignment-based methods such as BLASTP and DIAMOND are invaluable for predicting protein homology and function, but these methods perform poorly with highly divergent, chimeric, or incomplete sequences and cannot classify truly “novel” proteins that do not possess any homologs in databases.^6^^,^^7^^,^^8^ Consequently, how can the information held in the dark proteome be unraveled? Considering the recent advances in artificial intelligence (AI) to solve complex problems and decipher patterns in large datasets, could AI support protein classification and allow the annotation of unknown proteins from sequence datasets? The answer is “yes.”

**AI as a solution**: AI-supported bioinformatics already accelerates large-scale proteomics workflows and data interpretation, from peptide sequence determination to end-to-end analysis of complex datasets. Harnessing the capabilities of large language models (LLMs), Nelson et al.^6^ take this a step further, showing how LLM-based generative AI can greatly outperform conventional methods in speed, accuracy, and recall of protein classification (i.e., identification of all proteins that belong to a class). Alongside systems such as FANTASIA,^8^ which leverages protein language models for high-performance functional annotation, LLM-based AI opens a route to ultimately interrogate the dark proteome, as discussed below.

**LA⁴SR framework in a nutshell**: with promising capabilities in logical inference, knowledge synthesis, and cross-domain generalization, LLMs are showing potential to interpret chemical or biological data.^7^^,^^8^^,^^9^ The framework developed by Nelson et al.^6^ relies on LLMs, specifically transformers (Mistral, nanoGPT, GPT-NeoX, ByT5, DistilRoBERTa, MiniLM, BLOOM-560m, and Pythia) and state-space models (S6 models from the Mamba framework). For training and performance evaluation, the authors constructed large-scale microbial genomics datasets comprising ∼77 million distinct protein sequences, including translated ORFeomes from 166 microalgal genomes across 10 phyla (Chlorophyta, Rhodophyta, Haptophyta, Cercozoa, Ochrophyta, Myzozoa, Euglenophyta, Chlorachniophyta, Streptophyta, and Chromerida). Models were trained on either terminal information (TI)-inclusive datasets (i.e., full-length natural sequences) or TI-free datasets (i.e., synthetic chimeras with terminal regions and gene boundaries missing or mixed up), providing a robust and complementary approach to evaluate the protein classification performance across lineages (i.e., generalizability). Importantly, the datasets generated were also combined with bacterial (TI-free and TI-inclusive), archaeal, and fungal (TI-inclusive only) contaminant sequences, which is essential for the models to accurately recognize the genetic makeup of algae and identify potential contamination in sequencing data. The resultant TI-inclusive and TI-free models showed similar protein classification performance. However, TI-free models achieved up to 10-fold inference/tokenization speedups and exhibited distinctly fewer position-dependent attribution profiles. Therefore, the TI-free models demonstrated strong protein classification performance while exhibiting impressive generalization capabilities, even when handling incomplete or noisy sequence data. This strong performance was further evidenced during validation using holdout sets comprising unseen sequences from the training taxa (i.e., data that was not used during the model training), data from new in-house assemblies from seen species (the model microalga *Chlamydomonas reinhardtii*), and clean and contaminated assemblies from unseen genera (including genomes published after the model was trained). The authors also implemented several tools (SHAP [Shapley Additive Explanations], HELIX [Hidden Embedding Layer Information Explorer], DeepLift LA⁴SR, and DMMP [Deep Motif Miner Pro]) to interpret and to improve confidence in predictions. These tools also allowed them to determine the mechanisms of sequence pattern learning (e.g., how specific amino acid residues, their patterns, and positional relationships influence model decisions). This critical capability shed light on the biological relevance of model outputs and enabled the identification of, for example, evolutionarily meaningful motifs across diverse taxa. This evidenced that interpretability tools are not merely diagnostic but can also uncover biological information. By revealing how models learn from sequence data (e.g., identifying conserved motifs or residue patterns), these tools bridge the gap between computational predictions and biological understanding.

**Performance and scope**: LA⁴SR processed queries in 0.0378 ± 0.0029 s on an A100 GPU, >10,000× faster than NCBI BLASTP^+^ and 82× faster than DIAMOND on identical query sets. Across complete algal proteomes, LA⁴SR provided near-complete protein classification coverage, with >99% of sequences receiving a model prediction vs. ∼35% hits with BLASTP/DIAMOND, and achieved high accuracy on held-out and unseen data (e.g., macro *F*_1_ score of 0.9173). Overall, by treating protein sequences as a form of language, the developed models within the LA⁴SR framework learned the syntax governing protein function. This enabled the inference of structural motifs, functional domains, and evolutionary relationships from seen and unseen sequences. This approach extends the capability of conventional alignment-based tools in terms of throughput, coverage, and classification of previously unannotated sequences, while conventional methods remain indispensable for fine-grained homology, domain curation, and phylogenetic inference.

However, as acknowledged by the authors, the framework remains constrained by the limited availability of biological sequence data for several key algal groups that are underrepresented in public databases (e.g., non-Chlorophyta and non-Bacillariophyta). Broadening the pool of experimental data used for model training is therefore crucial to further enhance model performance and reliability.

The LA^4^SR framework, including all models and tools, is distributed in a Singularity container freely available for academic use, making it efficient, customizable, and accessible. The development of computationally efficient architectures and training strategies is a critical step toward democratizing the use of LLMs across the broader scientific community.^6^^,^^7^^,^^8^ Customizable and generalizable, the framework can be extended to additional taxa or organisms, deepening our understanding of biomolecular structure, function, and evolution. More specifically, microalgae such as the model *C. reinhardtii* offer a robust model system for elucidating mechanisms of environmental adaptation, including conserved mechanisms that are shared across both animals and plants.^10^ In combination with richer datasets, advanced language-model frameworks such as LA^4^SR should enable the systematic exploration of unknowns such as the dark proteome and the discovery of new metabolic pathways or regulatory mechanisms. The deeper understanding gained through these tools should guide the development of advanced molecular and genetic tools for fundamental research or biotechnological applications (e.g., bioengineering). Characterizing proteins with novel properties could therefore lead to the development of new biotechnological opportunities.

LA⁴SR thus represents an important step toward scalable and interpretable protein annotation in algae, with substantial potential for future developments and applications.

## Declaration of interests

The authors declare no competing interests.

## References

[1] Callaway E. (2025). ‘Dark proteins’ hiding in our cells could hold clues to cancer and other diseases. Nature.

[2] Pennisi E. (2024). ‘Dark proteome’ survey reveals thousands of new human genes. Science.

[3] Wright B.W., Yi Z., Weissman J.S., Chen J. (2022). The dark proteome: Translation from noncanonical open reading frames. Trends Cell Biol..

[4] Perdigão N., Heinrich J., Stolte C., Sabir K.S., Buckley M.J., Tabor B., Signal B., Gloss B.S., Hammang C.J., Rost B. (2015). Unexpected features of the dark proteome. Proc. Natl. Acad. Sci. USA.

[5] Pavlopoulos G.A., Baltoumas F.A., Liu S., Selvitopi O., Camargo A.P., Nayfach S., Azad A., Roux S., Call L., Ivanova N.N. (2023). Unraveling the functional dark matter through global metagenomics. Nature.

[6] Nelson D.R., Jaiswal A.K., Ismail N.S., Mystikou A., Salehi-Ashtiani K. (2025). Pan-microalgal dark proteome mapping via interpretable deep learning and synthetic chimeras. Patterns.

[7] Madani A., Krause B., Greene E.R., Subramanian S., Mohr B.P., Holton J.M., Olmos J.L., Xiong C., Sun Z.Z., Socher R. (2023). Large language models generate functional protein sequences across diverse families. Nat. Biotechnol..

[8] Martínez-Redondo G.I., Perez-Canales F.M., Carbonetto B., Fernández J.M., Barrios-Núñez I., Vázquez-Valls M., Cases I., Rojas A.M., Fernández R. (2025). FANTASIA leverages language models to decode the functional dark proteome across the animal tree of life. Commun. Biol..

[9] Chen X., Tang H. (2025). Designing a large language model for chemists. Patterns.

[10] Osnato M. (2021). Comparative genomics in *Chlamydomonas*: understanding the past, envisioning the future. Plant Cell.

